# Elevated Ambient Air Zinc Increases Pediatric Asthma Morbidity

**DOI:** 10.1289/ehp.10759

**Published:** 2008-02-07

**Authors:** Jon Mark Hirshon, Michelle Shardell, Steven Alles, Jan L. Powell, Katherine Squibb, John Ondov, Carol J. Blaisdell

**Affiliations:** 1 Department of Emergency Medicine, and; 2 Department of Epidemiology and Preventive Medicine, University of Maryland School of Medicine, Baltimore, Maryland, USA; 3 Division of Disease Control, Department of Public Health, Philadelphia, Pennsylvania, USA; 4 Department of Chemistry, University of Maryland, College Park, College Park, Maryland, USA; 5 Department of Pediatrics, University of Maryland School of Medicine, Baltimore, Maryland, USA

**Keywords:** air pollution, asthma, children, emergency departments, hospitals, zinc

## Abstract

**Background:**

Recent studies indicate that the composition of fine particulate matter [PM ≤ 2.5 μm in aerodynamic diameter (PM_2.5_)] is associated with increased hospitalizations for cardiovascular and respiratory diseases. The metal composition of PM_2.5_ influences allergic and/or inflammatory reactions, and ambient zinc contributes to worsening pulmonary function in susceptible adults. However, information is limited concerning associations between ambient air zinc levels and health care utilization for asthma, especially among children.

**Objective:**

We aimed to investigate the relationship between outdoor ambient air PM_2.5_ zinc levels and urgent health care utilization for children living in an urban area.

**Methods:**

We used a time-series study to estimate the association of ambient air PM_2.5_ zinc levels with hospital admissions and emergency department (ED) utilization by children in Baltimore, Maryland, controlling for time trends. We used data from daily discharge administrative claims of ED and hospital utilization for asthma in children, 0–17 years of age for Greater Baltimore from June 2002 through November 2002. We collected ambient air PM_2.5_ metal concentration data, determined by X-ray fluorescence spectroscopy, during the U.S. Environmental Protection Agency–sponsored Baltimore Supersite project.

**Results:**

Previous-day medium levels of zinc (8.63–20.76 ng/m^3^) are associated with risks of pediatric asthma exacerbations that are 1.23 (95% confidence interval, 1.07–1.41) times higher than those with previous-day low levels of zinc (< 8.63 ng/m^3^) after accounting for time-varying potential confounders.

**Conclusion:**

Results suggest that high ambient air PM_2.5_ zinc levels are associated with an increase in ED visits/hospital admissions for asthma on the following day among children living in an urban area.

Since the creation of the U.S. Environmental Protection Agency (EPA) with the Clean Air Act of 1970 (Clean Air Act Amendments 1970), understanding of the composition, sources, and effects of air pollution has greatly expanded. The 1990 Clean Air Act (Clean Air Act Amendments 1990) listed 189 hazardous air pollutants that the U.S. EPA now must evaluate to determine what regulatory action, if any, is needed. Among these many pollutants, there is increasing recognition that fine particles [particulate matter ≤ 2.5 μm in aerodynamic diameter (PM_2.5_)] are associated with cardiopulmonary health effects and excess morbidity and mortality (Coyle et al. 2006; Dominici et al. 2006; Miller et al. 2007). PM_2.5_ is derived from both natural and anthropogenic sources. Atmospheric zinc, for example, comes from incinerators, motor vehicles, and industry (Suarez and Ondov 2002). However, the health effects on humans of many of these airborne metals are poorly understood, and there is limited knowledge concerning the specific role that individual metal pollutants play.

Asthma causes significant morbidity, especially in children. Many studies have sought to determine environmental exposures that exacerbate asthma. The first evidence for a potential role of zinc in asthma exacerbations came from reports of zinc oxide exposure as a cause of occupational asthma in individuals working with heated zinc (Gordon and Fine 1993; Malo et al. 1993; Weir et al. 1989). In addition, animal studies indicated that metals such as zinc in ambient air PM_2.5_ samples cause acute pulmonary toxicity and increase the severity of allergic respiratory disease in rodents (Dye et al. 2001; Gavett et al. 2003). In humans, ambient air zinc and iron have been associated with worsening pulmonary function tests in susceptible individuals (Lagorio et al. 2006). In addition, some literature indicates that pulmonary toxicity from atmospheric dust samples can be attributed to zinc and copper (Adamson et al. 2000; Prieditis and Adamson 2002).

The use of emergency departments (EDs) and the need for hospitalization for asthma can be viewed as health outcome proxies for severity of respiratory disease. Claiborn et al. (2002) reported from Spokane, Washington, evidence of a relationship between ambient PM_2.5_ and health outcomes such as ED utilization. In particular, their findings suggest an association between ED visits for asthma and increased combustion products, air stagnation (which was associated with incomplete combustion products), and fine-particle zinc. To better understand the effect of ambient zinc on asthmatic children, we analyzed air pollution data from the Baltimore, Maryland, metropolitan area with ED utilization and hospitalization data for asthmatic children, to determine the relationship between ambient air zinc concentrations and asthma outcomes.

## Materials and Methods

### Data sources

#### Health services utilization data

**Figure 1:**
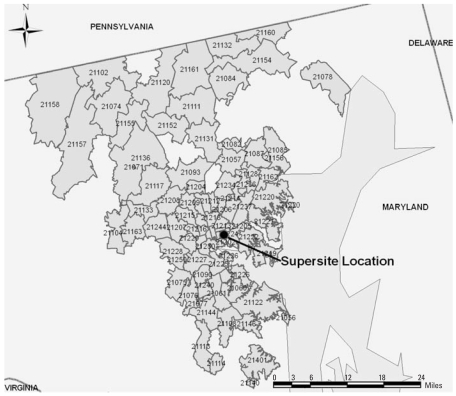
Baltimore Supersite location and Baltimore metropolitan-area ZIP codes used in analysis, 2005. Of note, because of changes in ZIP codes over time, this map represents approximate geographic coverage during the study period.

This is a time-series study of ED and hospital utilization for asthma by children living in the Greater Baltimore area. This area has a population of approximately 2,655,700, with 631,366 in Baltimore City in 2006. As previously described (Hirshon et al. 2006), the Health Care Services Cost Review Commission (HSCRC) of the Maryland Department of Health and Mental Hygiene collects service data from all Maryland, nonfederal hospitals. The HSCRC has two databases, one for ED visits resulting in discharges, and one for all hospitalized patients regardless of source of admission. Use of these databases primarily identifies individuals with significant asthma exacerbations. We obtained daily nonconfidential ED utilization and admission records for children, 0–17 years of age, for services rendered from June 2002 through November 2002. Utilization data for children who resided in the Greater Baltimore area (ZIP codes 21056 through 21251 and 21401) were included in the study, as shown in Figure 1. Asthma ED visits and hospitalizations were defined as any discharge with an ICD-9CM [*International Classification of Diseases, 9th Revision: Clinical Modification, 6th edition* (ICD-9CM 2001)] code with the first three digits of 493. The University of Maryland, Baltimore, Institutional Review Board approved this study.

#### Ambient air PM_2.5_ metal concentration and atmospheric data

PM_2.5_ were collected at the Baltimore “Supersite” at Ponca Street in East Baltimore (latitude 39.29°N, longitude 76.55°W) using a Speciation Trends Network sampler, as previously described (Ondov et al. 2006). The PM data represent 24-hr averages. The metals data used in the study were those determined by the U.S. EPA’s Speciation Trends Network program (U.S. EPA 2006), which normally reports results for samples collected every 6th day. In the Baltimore Supersite Study, the collection of samples was operated on the standard schedule except for two approximately 1-month periods (3 July 2002–15 August 2002 and 1 November 2002–30 November 2002) when daily sample collections and analyses were made. The samples were analyzed for elemental constituents by X-ray fluorescence by the U.S. EPA’s Speciation Trends Network contract laboratory (RTI International, Research Triangle Park, NC). Weather data were purchased from the National Oceanic and Atmospheric Administration.

### Data analysis

We combined deidentified data from the outpatient (ED) and inpatient HSCRC databases into a single database, matching entries on four independent variables to eliminate duplicates. The final data set represented a daily time series, by ZIP code, of number of pediatric ED visits or hospitalizations for asthma. This database was then merged with the air pollution data and analyzed using R software, version 2.4.0 (www.r-project.org).

Overdispersed Poisson mixed-effects regression models with a log link were used to estimate the association between zinc and daily counts of urgent health care utilization. Zinc levels were divided into categories defined by tertiles (low: < 8.63 ng/m^3^; medium: 8.63–20.76 ng/m^3^; high: > 20.76 ng/m^3^), because exploratory analysis revealed a nonlinear association between zinc levels and log-number of visits, a marked outlier (174 ng/m^3^), and no consensus exists in the literature for differentiating zinc values into low- or high-risk levels. Tertiles were used for parsimony of statistical modeling and interpretability. We included random effects for the intercept and zinc slopes to account for heterogeneity among ZIP codes, including spatial distance from the pollution monitor and demographic variation. Long-term, seasonal, and daily trends (i.e., weekend/ weekday), weather (temperature, barometric pressure, and precipitation), and other pollutants (nickel, chromium, iron, sulfate, ozone, carbon monoxide, elemental carbon, nitrogen dioxide, iron, and PM_2.5_) were explored as potential confounders using natural cubic splines. Degrees of freedom (df) of the splines for each covariate were chosen by comparing an adaptation of Akaike’s Information Criterion (AIC) between competing models, accounting for estimation of the overdispersion parameter, as in Kelsall et al. (1997). We estimated the autocorrelation function (ACF) of model residuals to assess whether observations were independent, given the model, because independence between ZIP codes over time is a key assumption of the model. We used this approach for inference, instead of generalized additive models (GAM) (Hastie and Tibshirani 1990), because multiple covariates were included in the model and small effects were expected—a scenario in which GAM may overestimate measures of association and underestimate standard errors (Dominici et al. 2002).

Three models were fit: one associating same-day zinc with ED/hospital visits, a 1-day lag model, and a 2-day lag model. The functional form of time trend was chosen based on AIC to control for unmeasured time-varying potential confounders. Given time trends, the best-fit function of weather and pollutants was chosen, again based on AIC. The same confounder model was used for all three lags of zinc.

We explored goodness of model fit by estimating the ACF of each model’s deviance residuals. We assessed the sensitivity of results to choice of potential confounders by comparing results to those from models including potential confounders that have an association with zinc, conditional on confounders included in the best-fit models. Last, we refit the models to include interaction terms between zinc and copollutants controlled for in the best-fit model. The copollutants were dichotomized at their median values, and associations of zinc with ED visits were estimated separately for days with high and low levels of copollutants, while controlling for continuous copollutants included in the best-fit model.

## Results

Using the outlined model-building strategy, the best-fit candidate model included natural cubic splines with degrees of freedom (df) = 3 for month and df = 4 for number of days since 1 June 2002. After controlling for time trends, ACF between consecutive days was reasonably low (no-lag, 0.17; 1-day lag, 0.17; 2-day lag, 0.17). Linear functions of elemental carbon, carbon monoxide, nitrogen dioxide, and iron were included in the model because they improved model fit, quantified by AIC. Summary statistics for zinc and asthma ED visits and hospitalizations (health care visits) are found in Table 1. Overall, there were 3,786 pediatric asthma ED visits and hospitalizations in Maryland during the 183-day study period by children residing in the Greater Baltimore area. During the 183-day period, the median number of daily urgent health care visits was 16 [interquartile range (IQR), 11–30].

Zinc was measured on 93 of the 183 days from 1 June 2002 to 30 November 2002, with a median (IQR) of 14.71 (7.53–25.30) ng/m^3^. The characteristics of children included in the models who visited EDs or were hospitalized are shown in Table 2. The number of visits included in the models were 1,813 (no-lag model), 1,819 (1-day lag model) and 1,784 (2-day lag model). The sex, race, and age distribution of the patients included in analyses did not vary by lag of zinc and did not vary over time (data not shown). Approximately 60% of visits included in analyses were made by males, 79% of visits were made by African Americans, and the most common age group presenting were school-age children (36% of visits). The random intercept accounted for 30% of the variation of health care utilizations after controlling for the variables included in the best-fit model. The random slopes for the effects of zinc accounted for < 1% of the variation of health care utilization.

Table 3 shows that, adjusted for time trends, same-day medium concentrations of ambient air PM_2.5_ zinc are associated with an increased risk of urgent health care utilization of 1.12 [95% confidence interval (CI), 0.98–1.28] times that on days with low levels of zinc (*p* = 0.09). Risk for asthma ED visits and hospitalizations for previous-day medium levels of zinc was 23% higher [relative risk (RR) = 1.23; 95% CI, 1.07–1.41] than for previous days with low levels of ambient zinc (*p* = 0.005). Same-day high levels of zinc have risks for asthma ED visits and hospitalizations that are 9% higher (RR = 1.09; 95% CI, 0.91–1.30) than same-day low levels of zinc. Previous-day high levels of zinc are associated with a risk for urgent health care that is 1.16 (95% CI, 0.97–1.39) times higher than that with previous-day low levels of zinc (*p* = 0.10). Risks of asthma health care utilization are 1.15 (95% CI, 0.96–1.38) times higher when 2-day lag zinc is high compared with when 2-day lag zinc is low. Last, the risk of asthma health care utilization is also elevated when 2-day lag zinc is at medium levels, compared with low levels (RR = 1.11; 95% CI, 0.94–1.30).

**Figure 2:**
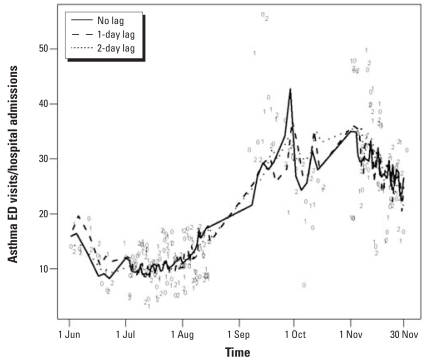
Asthma health care visits/admissions by lag and level of zinc, Baltimore, Maryland, 1 June 2002–30 November 2002. The number of ED visits and admissions are shown using all three lag models, with each line representing the estimates from the different models. 0, data point used in no-lag model; 1, data point used in 1-day lag model; 2, data point used in 2-day lag model.

**Figure 3:**
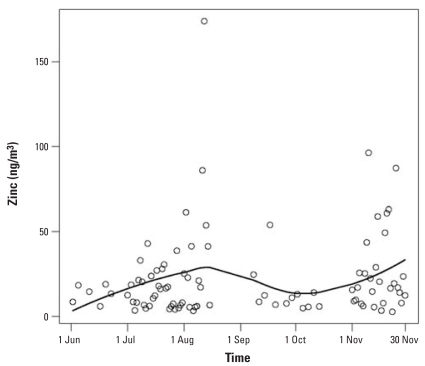
Zinc levels over time with LOESS smoother, Baltimore, Maryland, 1 June 2002–30 November 2002. The levels of zinc suggest peaks in August and November and troughs in June and October.

The number of ED visits and admissions over time on days included in the analyses and their estimated trend using all three lag models are displayed in Figure 2, showing lower numbers of visits, on average, in the summer than in the fall; the models fit the data well. Each line in Figure 2 represents the estimates from the different models. The levels of zinc and their estimated time trend fit with a LOESS smoother are shown in Figure 3, suggesting peaks in August and November and troughs in June and October; however, this is partially attributable to large outliers in August and November.

Air pollution is composed of many constituents. During the period of study, we found relatively strong correlations between zinc and other pollutants including nickel, chromium, iron, carbon monoxide, elemental carbon, and nitrogen dioxide, but relatively weak correlation between zinc and sulfate and ozone (Table 4), even after accounting for time trends. Also, a moderate correlation between zinc and temperature was found*.* After accounting for elemental carbon, carbon monoxide, iron, and nitrogen dioxide (the pollutants included in the best-fit Poisson regression model), the correlations between zinc and the remaining pollutants are attenuated. A sensitivity analysis was conducted that involved refitting each lag model excluding copollutants (including time trends only) and including nickel and chromium in addition to time trends, nitrogen dioxide, elemental carbon, carbon monoxide, and iron (Table 5). Except for same-day and 1-day lag models comparing high to low levels of zinc, the results show little sensitivity to choice of copollutants included in the models.

**Figure 4:**
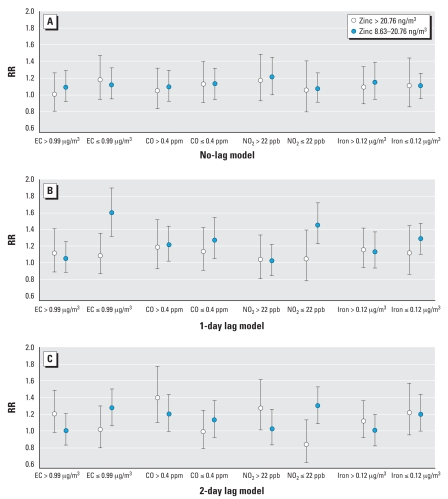
RRs (95% CIs) for high and medium levels of zinc (vs. low levels), separately for days with high (> median) and low (≤ median) levels of co-pollutants, Baltimore, Maryland, 1 June 2002–30 November 2002. Asthma ED visits and hospitalizations did not depend on the values of copollutants when comparing high and medium levels of same-day zinc to low levels of same-day zinc. However, the 1-day and 2-day lag models showed evidence of interaction of elemental carbon (EC) and nitrogen dioxide with zinc, and the 2-day lag model showed evidence of an interaction of carbon monoxide with zinc.

Results assessing interactions of copollutants elemental carbon, carbon monoxide, nitrogen dioxide, and iron with zinc are shown in Figure 4. The data showed little evidence that the RRs of asthma ED visits and hospitalizations comparing high and medium levels of same-day zinc to low levels of same-day zinc depend on values of copollutants (*p* for interaction > 0.20). However, the 1-day and 2-day lag models showed evidence of interaction of elemental carbon and nitrogen dioxide with zinc (p for interaction < 0.01). In both cases, the RR of asthma health care utilization comparing medium zinc to low zinc is higher on days with low levels of elemental carbon (≤ 0.99 μg/m^3^) and nitrogen dioxide (≤ 22 ppb). Last, the 2-day lag model showed evidence of an interaction of carbon monoxide with zinc (*p* for interaction = 0.048). The RR of asthma health care utilization comparing high zinc to low zinc levels is higher on days with high levels of carbon monoxide (> 0.4 ppm).

## Discussion

In this study, we explored the relationship between ambient-air zinc, a specific component of PM_2.5_, and the need for children to visit an ED or to be admitted to a hospital for asthma. Exposure to airborne zinc in PM_2.5_ increasingly appears to be associated with adverse health effects. Using urgent health care utilization data for asthma, we investigated the potential impact of ambient-air zinc on children. Risk of health care utilization for pediatric asthma is > 20% higher on the day after increased ambient zinc levels than on the day after low zinc levels. Our results are consistent with other published studies that focused primarily on respiratory or cardiovascular diseases in adults, only on ED visits, or did not differentiate the specific components of the fine particulate matter (Babin et al. 2007; Claiborn et al. 2002; Dominici et al. 2006; Miller et al. 2007).

The relative toxicity of the components found in PM_2.5_ requires further investigation, though epidemiologic research indicates that specific metals such as zinc, iron, copper, and nickel may disproportionately contribute to disease burden (Burnett et al. 2000). Intratracheal exposure studies of type 1 alveolar epithelial cells (Adamson et al. 2000) and studies in mouse lung (Prieditis and Adamson 2002) indicate that zinc is a significant toxic component of atmospheric particles leading to lung injury and inflammation. This may be related to the release of a number of proinflammatory cytokines, because recent research indicates that zinc oxide produces potent but reversible pulmonary inflammation (Sayes et al. 2007). However, further research is needed to determine the underlying mechanism of pulmonary toxicity from inhaled zinc.

Recent studies increasingly show significant adverse health effects of PM_2.5_ (Coyle et al. 2006; Dominici et al. 2006; Laden et al. 2006; Miller et al. 2007). PM_2.5_ is composed of a heterogeneous group of particles found in ambient air. These particles also contain metals from various sources, including industrial and automotive combustion. Previous publications related to occupational exposure to zinc fumes have shown clinical disease from exposure to significantly elevated levels of ambient zinc, for example, at 0.26–0.29 mg/m^3^ (Malo et al. 1993). Our results estimate elevated risks of health-care visits at the much lower level of ≥ 8.63 ng/m^3^, which may be attributable partly to the large sample size in this population based study. More recent research has begun to explore the relationship between health care utilization and components of PM_2.5_ including zinc (Claiborn et al. 2002; Frampton et al. 1999). Additional studies are needed to improve our understanding of the relationship between specific ambient metal levels and respiratory diseases to better direct regulatory action for protection of the public’s health.

Air pollution clearly contributes to the burden of respiratory, cardiovascular, and other human health problems (D’Amato et al. 2001; Heinrich et al. 2000). One disease that man-made fumes are known to adversely affect is asthma (Kuo et al. 2002; Peden 2001, 2002). Asthma is a chronic medical condition that can have acute, and at times life-threateningly severe, exacerbations. Both children and adults are affected by asthma, a significant clinical and public health problem in the United States (Mannino et al. 2002; U.S. Department of Health and Human Services 2000). Particularly in children, asthma morbidity has worsened in the United States and other countries over the past decades. Pollution not only contributes to acute exacerbations of asthma, but has also been implicated as a factor causing new cases of asthma (McConnell et al. 2002). Some of the main criteria pollutants are known to contribute to airway inflammation and to asthma exacerbations (Peden 2001). However, there are many other constituents to air pollution whose contribution to asthma disease burden is not well known. Of particular interest is the potential positive synergy, or interaction, of PM_2.5_ components in contributing to acute asthma morbidity. In this study, we found conflicting evidence of synergy between zinc and copollutants. We found positive synergy of high levels of zinc with carbon monoxide and nitrogen dioxide in the 2-day lag model. However, our estimated association in the 1-day and 2-day lag models of medium levels of zinc with asthma ED visits was higher when elemental carbon and nitrogen dioxide were low, suggesting a negative synergy. One plausible explanation for this result is that those whose asthma symptoms would be exacerbated by small elevations of zinc are those whose symptoms would be exacerbated by high levels of copollutants, thus leading to a negligible marginal increase in asthma ED visits with medium versus low levels of zinc when levels of copollutants are high. Further empirical studies examining synergy are needed to assess this hypothesis.

A causal link between air pollution and ED visits/hospitalizations cannot be established with the results of this study; however, we have found that medium and high levels of zinc are associated with increased acute care for children with asthma, after controlling for other criteria pollutants known to affect asthma outcomes. In the absence of personal exposure measurements, we assumed that additional time-varying individual-level zinc exposure undetected by the Supersite monitor does not confound time-varying ambient zinc detected by the monitor. For example, a child’s parents do not smoke more indoors in front of the child (source of zinc likely not detected by the monitor) on days where there are more industrial emissions (source of zinc likely detected by the monitor). It is possible that zinc is a surrogate of a component in outdoor air that we did not analyze and that is a key trigger of asthma exacerbations. We were also not able to control for other exposures such as second-hand cigarette smoke, which is known to contain zinc. We recognize that administrative data must be used cautiously when conducting research, because the primary uses of these data are for nonresearch purposes. Although severity of disease clearly influences the need to seek care for acute-exacerbation asthma, many other factors influence the decision to seek medical care aside from air quality, including socioeconomic status (Babin et al. 2007). Only the primary diagnosis code was used for analysis, so asthma cases may have been underestimated throughout the time period, but this should not be differentially affected by high or low days of ambient zinc. Postal codes of residence were used to identify individuals who required a visit to a health care facility. Though unlikely, health care visits may have occurred outside Maryland by Baltimore metropolitan-area residents. One of the limitations of the zinc concentration data is that PM_.25_ pollutants were collected inconsistently from a single site. This was attributable to the expense of analyzing continuously such a large number of air pollution components. Despite these limitations, there was no sampling bias for selection of days for measuring metal levels and corresponding health utilization, because days for measurement were not chosen based on levels of ED visits or anticipated levels of metals (Rubin 1976).

## Conclusion

Results suggest that days with increased ambient zinc levels are associated with an increased risk of ED visits/hospital admission for asthma on the following day among children living in the Greater Baltimore area. Additional analyses suggested that this relationship is not sensitive to inclusion of other copollutants in the model, and that zinc may have synergistic effects with carbon monoxide, elemental carbon, and nitrogen dioxide, although further study is needed.

## Figures and Tables

**Table 1:** Summary statistics for zinc and ED visits/hospital admissions for children, Baltimore, Maryland, 1June 2002–30 November 2002.

Parameter	Zinc (ng/m^3^)	Asthma ED visits/hospital admissions
No. of days	93	183
Mean	22.42	21
SD	25.14	12
Lower quartile	7.53	11
Median	14.71	16
Upper quartile	25.30	30

**Table 2:** Characteristics of children hospitalized or who visited the emergency department for asthma, Baltimore, Maryland, 1 June 2002–30 November 2002 [no. (%)].

Characteristic	Days with zinc collected	One day after those with zinc collected	Two days after those with zinc collected
Sex			
Male	1,093 (60)	1,120 (62)	1,094 (61)
Female	720 (40)	699 (38)	690 (39)
Race/ethnicity			
Caucasian	353 (19)	357 (20)	355 (20)
African American	1,430 (79)	1,433 (79)	1,398 (78)
Other	30 (2)	29 (1)	31 (2)
Age (years)			
Infant (0– < 1)	104 (6)	101 (6)	106 (6)
Toddler (1– < 3)	352 (19)	367 (20)	363 (20)
Preschooler (3– < 6)	391 (22)	402 (22)	373 (21)
School-age (6– < 13)	659 (36)	653 (36)	641 (36)
Adolescent (13– < 18)	307 (17)	296 (16)	301 (17)

**Table 3:** RR (95% CI) and *p*-values for zinc, Baltimore, Maryland, 1 June 2002–30 November 2002.

	Best-fit model^a^					
	No lag		1-day lag		2-day lag	
Zinc level (ng/m^3^)	RR (95% CI)	*p*-Value	RR (95% CI)	*p*-Value	RR (95% CI)	*p*-Value
Low (< 8.63)	(referent)	—	(referent)	—	(referent)	—
Medium (8.63–20.76)	1.12 (0.98–1.28)	0.09	1.23 (1.07–1.41)	0.005	1.11 (0.94–1.30)	0.21
High (> 20.76)	1.09 (0.91–1.30)	0.32	1.16 (0.97–1.39)	0.10	1.15 (0.96–1.38)	0.13

a Overdispersed poisson mixed-effects regression controlling for natural cubic spline terms for month (df = 3) and number of days since 1 June 2002 (df = 4) and the pollutants elemental carbon, carbon monoxide, nitrogen dioxide, and iron fixed effects and a random intercept and zinc effects. PM_2.5_ and temperature were excluded because inclusion resulted in non-convergence owing to multicollinearity. Chromium and nickel did not contribute to model fit as measured by an adaptation of the AIC.

**Table 4:** Pearson’s correlation coefficient between zinc and other candidate pollutants and weather variables, Baltimore, Maryland, 1 June 2002–30 November 2002.

Potential confounder	Correlation with zinc	Adjusted correlation with zinc (model 1)^a^	Adjusted correlation with zinc (model 2)^a^
Nickel^b^	0.41	0.39	0.09
Chromium^b^	0.17	0.24	0.12
Iron^b^	0.54	0.52	—
Sulfate	0.01	−0.01	−0.04
Carbon monoxide^b^	0.40	0.47	—
PM_2.5_^b^	0.39	0.47	0.24
Ozone	0.01	0.12	0.20
Nitrogen dioxide^b^	0.66	0.63	—
Elemental carbon^b^	0.48	0.53	—
Barometric pressure (mmHg)	0.07	0.11	−0.02
Temperature^b^ (°F)	0.03	0.28	0.11
Precipitation (inches)	−0.08	−0.06	−0.11

a Residuals from linear regression models, after accounting for time trends (3 df for month, 4 df for days since 1 June 2002) (model 1), and after controlling for time trends, elemental carbon, carbon monoxide, nitrogen dioxide, and iron.

b Explored as potential confounders for association between zinc and health care utilization.

**Table 5:** RR (95% CI) and *p*-values for sensitivity analysis models of zinc and potential confounders, Baltimore, Maryland, 1 June 2002–30 November 2002.

	Sensitivity analysis models					
	Controlling for time trends only^a^			Controlling for time trends and additional copollutants^b^		
Zinc level (ng/m^3^)	No lag	1-day lag	2-day lag	No lag	1-day lag	2-day lag
Low (< 8.63)	(referent)	(referent)	(referent)	(referent)	(referent)	(referent)
Medium (8.63–20.76)	1.08 (0.95–1.23)	1.13 (1.003–1.28)	1.13 (0.98–1.31)	1.12 (0.98–1.29)	1.20 (1.04–1.38)	1.12 (0.95–1.32)
High (> 20.76)	0.98 (0.86–1.11)	1.03 (0.91–1.16)	1.15 (1.01–1.30)	1.09 (0.91–1.31)	1.12 (0.93–1.35)	1.19 (0.98–1.44)

a Overdispersed poisson mixed-effects regression controlling for natural cubic spline terms for month (df = 3) and number of days since 1 June 2002 (df = 4) fixed effects and a random intercept and zinc effects.

b Overdispersed poisson mixed-effects regression controlling for natural cubic spline terms for month (df = 3) and number of days since 1 June 2002 (df = 4) and the pollutants elemental carbon, carbon monoxide, nitrogen dioxide, iron, nickel, and chromium fixed effects and a random intercept and zinc effects.
